# How young children learn independent asthma self-management: a qualitative study in Malaysia

**DOI:** 10.1136/archdischild-2019-318127

**Published:** 2020-07-03

**Authors:** Siti Nurkamilla Ramdzan, Ee Ming Khoo, Su May Liew, Steven Cunningham, Marilyn Kendall, Nursyuhada Sukri, Hani Salim, Julia Suhaimi, Ping Yein Lee, Ai Theng Cheong, Norita Hussein, Nik Sherina Hanafi, Azainorsuzila Mohd Ahad, Hilary Pinnock

**Affiliations:** 1 Usher Institute of Population Health Sciences and Informatics, The University of Edinburgh, Edinburgh, UK; 2 Department of Primary Care Medicine, Faculty of Medicine, University of Malaya, Kuala Lumpur, Malaysia; 3 Department of Family Medicine, Universiti Putra Malaysia Faculty of Medicine and Health Sciences, Serdang, Selangor, Malaysia; 4 Lukut Health Clinic, Ministry of Health Malaysia, Putrajaya, Wilayah Persekutuan, Malaysia

**Keywords:** asthma, children, qualitative research, general paediatrics, patient perspective

## Abstract

**Objective:**

We aimed to explore the views of Malaysian children with asthma and their parents to enhance understanding of early influences on development of self-management skills.

**Design:**

This is a qualitative study conducted among children with asthma and their parents. We used purposive sampling and conducted focus groups and interviews using a semi-structured topic guide in the participants’ preferred language. All interviews were audio-recorded, transcribed verbatim, entered into NVivo and analysed using a grounded theory approach.

**Settings:**

We identified children aged 7–12 years with parent-reported, physician-diagnosed asthma from seven suburban primary schools in Malaysia. Focus groups and interviews were conducted either at schools or a health centre.

**Results:**

Ninety-nine participants (46 caregivers, 53 children) contributed to 24 focus groups and 6 individual interviews. Children mirrored their parents’ management of asthma but, in parallel, learnt and gained confidence to independently self-manage asthma from their own experiences and self-experimentation. Increasing independence was more apparent in children aged 10 years and above. Cultural norms and beliefs influenced children’s independence to self-manage asthma either directly or indirectly through their social network. External influences, for example, support from school and healthcare, also played a role in the transition.

**Conclusion:**

Children learnt the skills to self-manage asthma as early as 7 years old with growing independence from the age of 10 years. Healthcare professionals should use child-centred approach and involve schools to facilitate asthma self-management and support a smooth transition to independent self-management.

**Trial registration number:**

Malaysian National Medical Research Register (NMRR-15-1242-26898).

## Introduction

With an estimated prevalence in Malaysia of 8.9% in schoolchildren, asthma is the the most common chronic disease among children and responsible for significant morbidity and mortality.^1–3^ Supported self-management is a key component of asthma management which improves asthma control and reduces asthma exacerbations.^4–6^ Self-management involves discussion of a personalised asthma action plan empowering children and their parents to identify triggers, recognise deteriorating control, and take timely and appropriate action.^4^

Despite strong recommendations from international asthma guidelines on the importance of supported self-management,^4 7^ and explicit promotion of asthma action plans by Malaysian guidelines since 1996,^8–10^ the concept of self-management is not widely understood in Malaysia.^11 12^ Compared with adults, the concept is more complex among children as many children with asthma are diagnosed at an age when parents are responsible for making all management decisions.^13^ Once at school and away from their parents for periods of time, children are expected to begin to learn to self-manage aspects of their own asthma.^14^ This transition is vitally important, as asthma may continue into adulthood^15^ and an understanding of how children learn to self-manage will inform effective delivery of supported self-management.

The process of transition to independent self-management of long-term conditions in children is part of normal development, but how this plays out is influenced by culture, beliefs and parenting style.^16–18^ Most studies have been conducted among adolescents in the USA, UK and European countries with very little research done among children in the Asian region.^16 19–22^ We therefore aimed to explore the perceptions of Malaysian children (aged 7–12 years) with asthma complemented by perceptions of their parents on the development of self-management skills. This will enhance understanding of the early influences on asthma self-management skills in children.

## Methods

We conducted a qualitative study among children with asthma and their parents in seven primary schools in Port Dickson, a suburban district of Malaysia. For simplicity, we use the term ‘parents’ to describe parents and other family carers, for example, grandparents.

### Context and school recruitment

Malaysia is a multicultural society with three main ethnic groups (Malay, Chinese and Indian). Between the age of 7 and 12 years, children typically attend a government primary school that teaches in one of the three languages according to parents’ choice.^23^ We conducted our study in the three main types of government primary schools (Malay, Chinese and Indian) to encompass the cultural diversity. The invitations, questionnaires, focus groups and interviews were conducted in the language preferred by the participants.

### Identifying and recruiting children with asthma

To identify children diagnosed with asthma, the participating schools sent letters enclosing screening questionnaires to the parents of all pupils asking whether their child had been diagnosed with asthma and had received any treatment for asthma in the past 12 months. If the answers were yes to both, we asked parents to complete a questionnaire on asthma control based on their child’s symptoms. The completed questionnaires were returned to the schools and we purposively sampled children with asthma from different age groups, ethnicities and degrees of asthma control. We invited parents of the selected children and their child to participate. Parents provided written consent for participation for themselves and their child. In addition, we obtained assent from the children.

### Data collection

We arranged focus groups or individual interviews according to participants’ preference. Parents and children’s participation were paired, but focus groups were carried out separately for parents and children to enable peer interaction. We collected basic sociodemographic data (online supplementary 1), and used a piloted topic guide (online supplementary 2) and pictures of asthma medications and treatments to stimulate discussion. All the interviews and focus groups were moderated by researchers who were native speakers of the language in which the session was conducted, and were carried out either at the child’s school or a health centre. The focus groups and interviews were audio-recorded, transcribed verbatim and analysed using NVivo software V.11.

10.1136/archdischild-2019-318127.supp1 Supplementary data

10.1136/archdischild-2019-318127.supp2 Supplementary data

### Language and translation

Tamil and Mandarin transcripts were translated to English by experienced bilingual English and native speakers of the source language. Bilingual moderators checked all English transcripts for semantic and contextual equivalence. The Malay transcripts were analysed in the source language, as researchers were fluent in Malay, the national language, and translated into English for publication.

### Data analysis

We used a constructivist grounded theory approach, a systematic but flexible technique, to analyse our findings.^24–26^ This uses an inductive, iterative and comparative approach to increase the explanatory and abstract level of emerging themes.^25 26^ The data from children and parents were analysed separately, and then compared between child–parent dyads. Three researchers (SNR, HS and NS) who are Malay native speakers coded the first Malay transcript independently. They read the transcripts word by word, with contextual information from field notes, and created codes to describe the meaning. They met to agree to a coding framework, which was then discussed with the team. SNR paired with one of the authors to code independently the remaining transcripts, guided by the framework. Any new codes were added to the framework in discussion with the team. SNR compared the codes, proceeded with focus and axial coding, and developed a schema by linking codes, subthemes and themes.^25^ To facilitate interpretation, we adopted the learning development categorisation used in the educational system^27^: children 7–9 years old are described as ‘young’; children 10–12 years old are ‘older’ children.

### Reflexivity and interpretation

The researchers most closely involved in data collection and coding were SNR (a Malay primary care physician, mother of a child with asthma), HS (a primary care physician) and NS (a Master’s student in health service research). All three are involved in research related to implementing supported self-management and, as long-term residents of Malaysia, are familiar with the local cultural beliefs, practices and parenting styles that could influence the self-management beliefs or practices of the participants. We remained aware of the importance of reflexivity, and interpretation involved regular discussions with a wider research team including primary care physicians and paediatricians from UK and Malaysia, and a social scientist, who was able to provide additional perspectives.

## Results

Between April and December 2016, 53 children participated in 12 focus groups (up to 9 participants; duration up to 100 min) and 5 individual interviews (duration up to 24 min). Parents (n=46) participated in 12 focus groups (up to 6 participants; duration up to 60 min) and 1 individual interview (duration 29 min). Two parents and two children were re-interviewed to clarify points from their first focus groups. Table 1 shows the characteristics of the participants.

**Table 1:** Characteristics of participants

Demographic characteristics of children	Malay n=16	Chinese n=10	Indian n=27
Age range (years)	10–12	9–12	7–12
Female	8	5	7
Male	8	5	20

Demographic characteristics of parents	Malay n=16	Chinese n=9	Indian n=21
Age range (years)	33–51	31–62	30–68
Female	16	9	17
Male	0	0	4
Educational background			
Primary	1	2	3
Secondary	9	7	9
Tertiary	6	0	1
Missing	0	0	8
Carer relationship with children			
Parents	16	8	19
Grandparents	0	1	2

### Summary of findings

**Figure 1:**
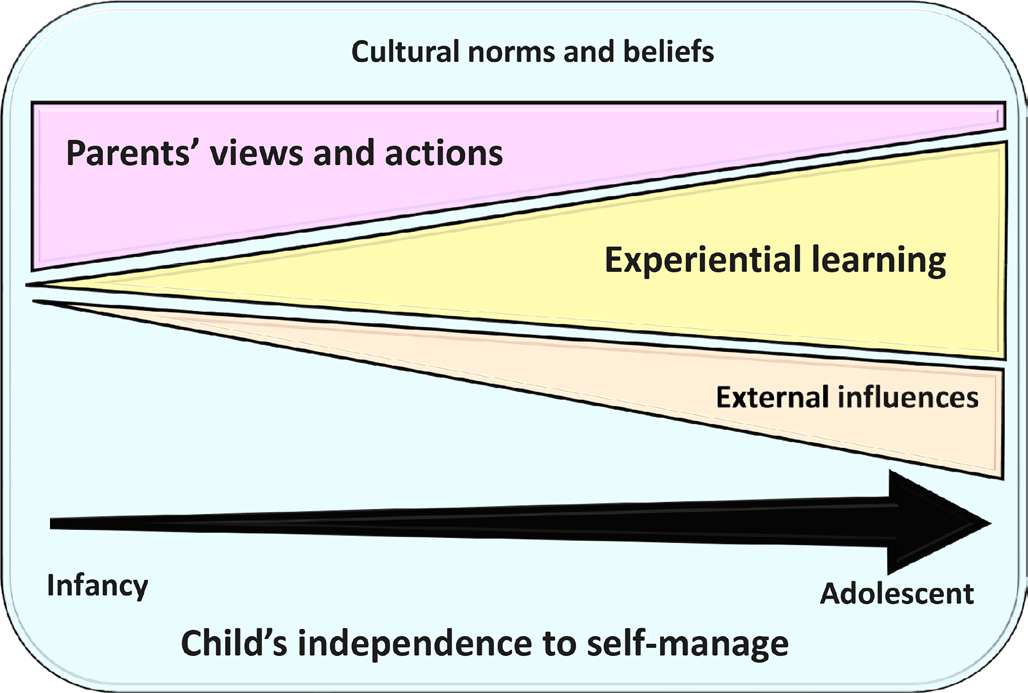
Schema of transition to independent self-management of care among children with asthma.

Figure 1 summarises our findings on transition to self-management among children with asthma. Additional illustrative quotes are in tables 2–4.

**Table 2:** Quotes illustrating the transfer of control

Acceptance of parents’ views and actions	
(a) Mirroring parents’ beliefs	“Drink hot water… cough will reduce… can breathe and will be better.” (D5_FGD5ICh, 10-year-old Indian boy) “If he continuously drinks the warm water, it can help to prevent getting phlegm (which causes asthma).” (K7_FGD7IC, Indian, mother of D5)
(b) Mirroring parents’ actions	“Take a lot of nutritious food like honey, and religiously encouraged food like pomegranate” (W2_FGD2MCh, 11-year-old Malay boy) “I think it is good to give supplement because I gave his pomegranate juice for quite some time and he did not have any attack.” (AM1_FGD1MMC, Malay mother to W2)
(c) Parental ‘rules‘	“I like cats and dogs. It’s fluffy and I like to touch and play with it. But then the fur and dust will be flying. When I breathe it gets into my nose. Will be sneezing a lot. That’s when I get asthma.” (NG3_FGD3ICh, 11-year-old boy) “I had dogs in my house. He (my son) had problem and had attacks when we had furry dogs. Now, the dogs will stay outside of the house.” (PK4_FGD4IC, Indian mother to NG3)
(d) Parental confidence in child’s ability to manage asthma	“I’ll drop by the school and give the inhalers. I wouldn’t let my child bring it along. I’ll just come on time, give the puff using the inhalers and let them go back to their class.” (KO4_FGD4IC, Indian, mother of a 10-year-old boy)
**Experiential learning and growing independence with age**	
“When I have asthma, I’ll scream and call my mum… Mom will drive me to hospital, and they’ll give gas.” (H6_FGD6_ICh, 7-year-old Indian girl)	
“Moderator: What do you do when you have asthma? PU1: My mum asks me to use the inhaler. Moderator: Take the inhaler, what do you do after that? PU1: If not better after using the inhaler, will go and get gas (nebuliser).” (FGD1_MCh, 10-year-old Malay girl)	
“AM2: Controlled. Moderator: You said it(asthma) is controlled although it disturbed you? AM2: Because now, I already know how to some breathing technique, no need to use the blue inhaler.” (AM2_FGD2MCh, 12-year-old Malay boy)	
“When I was late for school, I ran to school and asthma sometimes occurred. That time, I would slow down and walk slowly, then the breathing discomfort would reduce… Sometimes I have to rest for 30 minutes and sometimes even one hour after intense exercises… I will wait by my self. Sometimes I will tell my mother after school if there was still breathing discomfort. Usually at school, I will figure out myself.” (C1_IDI4_CCh, 12-year-old Chinese boy)

Box 1 **Quotes illustrating cultural norms and beliefs**

- “They (parents) say not to eat mutton. It causes runny nose and causes (which leads to) asthma. When there is dust, or house cleaning they suggest me to wear mask (so I don’t get runny nose).” (NR3, FGD3_ICh, 11-year-old Indian boy)
- “So, they get runny nose and cough very frequent and it eventually will cause them to have asthma. I tried changing the milk powders for them, but still they’ll get runny nose. They get very bad runny nose and later on it led to asthma.” (PK4, FGD4_IC, Indian, mother of NR3)
- “Doctors do not allow us to do vigorous exercise and advised mom not to give us to drink those icy things.” (P1_FGD2_CCh, 12-year-old Chinese boy)

Box 2 **Quotes illustrating external influences**

**School influences**

- “Moderator: What will happen if you use inhaler in front of your friends?
- WA: My friends do not laugh at me. Sometimes they want to try.” (FGD6_MCh, 12-year-old Malay boy)
- “If it is too bad, my teacher will call my mom. But if the teacher asked me and I am okay, then I will go back to class.” (FA2_FGD2MCh, 12-year-old Malay girl)

**Healthcare influences**

- “Moderator: When you attend the clinic, do you have bad feelings or your feel okay?
- SA5: I like
- NA5: I don’t mind
- AD5: I like
- Moderator: You like to attend, why do you like it?
- AD5: because he has asthma” (FGD5_MC, SA5, 10-year-old Malay boy; NA5, 10-year-old Malay boy; and AD5, 11-year-old Malay boy)
- “We went to the clinic few times but I did not understand what the doctor said.” (IDI1_CCh, 9-year-old Chinese boy)

### Transfer of control

#### Acceptance of parents’ views and actions

Parents were the main source of information for asthma and asthma management; their influence was notably stronger among younger children than older children (table 2). Younger children’s understanding of asthma was almost exclusively based on their parents’ beliefs and actions. An example was the belief that ‘hot and cold’ are important as cause, trigger and treatment for asthma.

“Cannot drink ice water, cannot take anything ‘cold’, cannot eat orange.” (SC2_ICh, 9-year-old lndian boy, child of A9)

“For my son, cool drinks and oranges. He started to have flu and then it became phlegm and eventually asthma. All because of taking ice water.” (A9_FGD 9_IC, Indian mother)

Children who believed in the use of warm water for the treatment of asthma had parents who gave warm water to prevent and reduce asthma symptoms. Similarly, children used evidence-based medicine for self-management of asthma if their parents believed and practised self-management as recommended by healthcare professionals.

“First you need to use the inhaler (if you have asthma symptoms) and if you do not get better, you need to go and get nebuliser.” (P1_FGD1MCh, 10-year-old Malay girl)

“I will give the inhaler first but if this is still not working, I will go (to seek medical attention) even if it is 1, 2 or 3 am…” (M1_FGD1MC, Malay, mother of P1)

Parents influenced their child’s self-management by imposing ‘rules’, for example, not allowing their child to participate in sports. Parents based these constraints on their past experiences of coping with their child’s asthma (especially if their children had more troublesome symptoms) and their health beliefs.

“After I ran one round, I will make some noise, a little bit.” (FF1, FGD1CCh, 12-year-old Chinese girl)

“Don’t do strenuous exercise, then she will have less asthma attack.” (MS1FGD1CC, Chinese, mother of FF1)

Parental confidence in their child’s ability to independently self-manage varied. Although the age of the child was a factor in this decision, there were examples of parents of 12-year-old children ‘rushing back and forth’ to school as well as children as young as 10 years old being trusted to self-medicate.

“I’ll pick him from school, bring him home in the middle of his class. If I’m working, I’ll have rush back and forth to work and school. (SF2_FGD2_IC, Indian, father of a 12-year-old boy)

“I will remind her to use the inhaler if she is going to play or she feels her chest is tight. Asked her to use the inhaler, she carries it to school.” (MA1_FGD1_MC, Malay, mother of a 10-year-old girl)

#### Experiential learning

Some older children described how they learnt about asthma based on their own experiences, gaining confidence in self-managing their asthma (table 2). Several children developed their own self-management practices by experimenting and gaining experience by ‘trial and error’. All children were aware of the constraints imposed by their parents to prevent asthma symptoms and attacks. However, children as young as 9 years old challenged their parents’ views and actions, secretly undertaking ‘banned’ activities that they enjoyed, although sometimes worried that they might be blamed for causing an asthma attack. Some parents were aware that their child did not adhere to the constraints but realised that they were not able to supervise their child all the time.

“P2: My mother usually does not allow us to eat ice cream.

Moderator: Not allowed you to eat ice cream. Why?

P2: Afraid that we will have an asthma attack.

P1: Afraid of asthma attack.

Moderator: So, do you usually eat or do not eat?

P1, P2 and P3: I do.

Moderator: You all have. So, your mother allows you to eat it or you eat it secretly?

P1 and P2: I eat it secretly

Moderator: After eating it, will you have asthma attack?

P1: No

P2: No if you eat a lot then you will have asthma attack.”

(FGD1_CCh, P1, 9-year-old Chinese girl; P2, 12-year-old Chinese boy; P3, 10-year-old Chinese girl)

“SC: Drink too much of ice water, wow it’s terrible, asthma attack will come immediately…. I’ll definitely stop him. But he occasionally will take ice water secretly. My house has a water filter, there are three types: hot, warm, and cold. He will secretly press the cold, then press hot, and then cold again….

SB: Sometimes you cannot monitor your child. She will go and drink ice secretly.”

(FGD 3_CC, SC; Chinese, mother of P1 and P2; SB, Chinese, mother of P3)

### Cultural norms and beliefs

Cultural norms and beliefs influenced the children’s self-management both directly through their parents and indirectly through the child’s social network (box 1). Parents imparted cultural norms to their children by sharing their understanding of asthma and administering complementary medicine. Some healthcare personnel supported the parents’ cultural beliefs (eg, hot and cold beliefs as the cause/trigger for asthma) which further influenced children self-management of asthma.

“For me, my doctor told me that I got asthma when I went to clinic. And then they told me not to drink ice water, not to play in the rain or play in cold water.” (S8, FGD8_ICh, 10-year-old Indian boy)

### External influences

Teachers, friends, healthcare professionals and other family members could influence the child’s transition to independent self-management (box 2). Negative attitudes (mocking inhaler use, ‘banning’ use of unfamiliar spacers) were potential barriers to confident self-management. In contrast, good support (sharing experience of having asthma with friends) appeared to have a positive influence on the transition.

“She brought it (inhaler and chamber) and her teacher scolded her, saying that she is playing. So, I had to call the school and say, it is not a toy. Maybe her teacher hasn’t seen a chamber before.” (MA2_FGD2_MC, Malay, mother of a 10-year-old girl)

#### School influences

Children and parents viewed school support for asthma self-management as limited. Some children as old as 11 years were dependent on others for decision-making. For example, they would inform their teachers whenever they had symptoms, who in turn contacted the parents to attend and administer an inhaler. In contrast, some younger children carried an inhaler and sought help only if they were unable to get relief themselves.

“Moderator: If at school and your mother is not around, what would you do if you have asthma (symptoms)?

AD5: Tell teacher and teacher will call mother and my mother will come.”

(AD5_FGD5_MCh, 11-year-old Malay boy)

“AS3: when my asthma is bad, I’ll bring the inhaler and spacer, and use it in class.” (AS3_FGD3MCh, 10-year-old Malay girl)

#### Healthcare influence

Children perceived little direct communication with healthcare professionals as most healthcare interactions were only with their parents. Discussions did not involve the children and no attempt was made to empower them to self-manage their asthma.

“They always talk to mum. I’ll be taking nebuliser, they’ll (mother and doctor) be talking there. I didn’t know what was happening.” (V6, FGD6_TCh, 9-year-old Indian boy)

## Discussion

The beginning of the transition to independence was detectable even in the younger children and was clearly apparent in older children. Younger children mirrored their parents’ actions and views. However, by the time they reached 10–12 years of age, they were learning from their own experiences, challenging parental constraints and gaining confidence to independently self-manage their asthma. All children were aware of their parents’ strategies to prevent asthma, but older children experimented and adapted their self-management practices. Cultural norms and beliefs influenced children’s transition to independence either directly or indirectly through the child’s social network. Schools and healthcare appeared to have a limited role in influencing the transition to independent self-management.

We conclude that self-management education should occur early as children can be expected to learn some degree of self-management once they start primary school, though clearly, young children are not capable of independent self-management. Indeed, even by the age of 15 years, some studies suggest that only three-quarters are capable of self-managing independently.^28^ There are also cultural differences as Asian parents tend to provide autonomy to their child at later age compared with non-Asians which may delay the transition to independence among Asian children.^18^ Healthcare professionals may need to guide parents in this process of transferring the responsibility to their child.^29 30^

Children’s own experiences in self-managing asthma appeared to be an important influencer in the transition to independent self-management. Our study highlighted that the children had different goals and developed different strategies to self-manage asthma compared with their parents, particularly when they were older. This is consistent with the findings of a US study that also found that primary school children and parents were not co-managing, but working independently, to self-manage asthma.^31^ Children frequently experimented to achieve their desired goals, often at school when their parents were not around. Similarly, a UK study identified that ‘trial and error’ was an important component in determining self-management decision-making among children.^32^ Another US study found that children presented unpleasant personal experiences, for example, bad taste of medicine or perceived lack of effectiveness of medication, as barriers to using treatment.^33^ This is contrary to the attitudes of healthcare professionals who tend to under-recognise children’s experience in self-management.^34^

Echoing the experience related by the children in this study, healthcare professionals typically only engaged with the parents in consultations, particularly when explaining management.^34 35^ International and national asthma guidelines recommended the involvement of children during education of asthma management; however, a child-centred approach in review consultations was not emphasised.^4 10^ Our finding that even young children are learning to self-manage their asthma suggests it is important for healthcare professionals to listen to children’s experiences and enquire about the strategies they used to achieve their desired goals. A more child-centred approach is likely to facilitate the child’s emerging self-management skills and improve asthma control.

The reports from our participants suggested that their healthcare professionals rarely communicated directly with the child. Self-management education and shared decision-making (even with adults) are not widely practised in Malaysia,^11 12^ reflecting traditional cultural norms as well as lack of training on communicating with patient/children dyads during clinical consultations.^36 37^ In addition, there are practical challenges in Malaysia (eg, lack of continuity of care) which make it more difficult to establish trusting relationships with children and their families.^3 38^

Parents are the primary decision-maker on the treatment of a child’s asthma,^33^ and their involvement in self-management is crucial as young children mirror their parents’ views and actions. However, a child-centred approach views care from the perspective of the child,^39 40^ providing developmentally appropriate care tailored to the individual child’s capacity, needs and motivation which evolve as the child grows.^39 41^ Clinicians need to work in partnership with parents and children to ensure safe management of asthma.^29 39^ Striking a balance between parental involvement, and re-directing the focus of consultation to the child, is a challenge for healthcare professionals.^18 40 42^

Support from school (and others in the child’s social network)^41^ could support transition to independent self-management for children with asthma. School-based interventions, sensitive to local cultural beliefs, could reduce stigma and promote awareness of asthma.^43–45^ School policies for managing asthma symptoms that arise during the school day could expect maturity-appropriate action on the part of the child as well as describing safe response from school staff.^6 41^

### Strengths and limitations of the study

This study explores the early stages of transition to independent self-management among primary school children. A strength is the exploration of the children’s perspectives on asthma self-management, matched to that of their parents, enabling triangulation. We involved a large number of participants with different cultural backgrounds conducted in their preferred language enabling cross-cultural comparison of practices and beliefs. A limitation is the identification of children based on parent-reported physician-diagnosed asthma without objective confirmation of the diagnosis. However, the participants’ perspective remains valid. Only 10 of the participants were between the ages of 7 and 9 years as this age group were more likely to be shy and declined assent to participate.

## Conclusion

Parents, school and healthcare professionals should be aware that asthma self-management skills emerge as young as 7 years of age in children across different cultures. Recognition that children experiment with their asthma management (sometimes unbeknown to their parents) offers opportunities for child-centred discussion. Healthcare professionals should proactively engage directly with children during consultations and involve them in management decisions from an early age. This has the potential to empower children to use asthma self-management skills that are commensurate with their developmental stage and to promote smooth and supported transition to independent self-management of their asthma.
