# Supplementary material for: How young children learn independent asthma self-management: a qualitative study in Malaysia
Source: Arch Dis Child. 2020 Jul 3;105(9):819–24. doi: 10.1136/archdischild-2019-318127 (PMC7456543; doi:10.1136/archdischild-2019-318127)
Supplement: Supplementary data [file archdischild-2019-318127supp001.pdf]

**Characteristics of participants (Carers/Child)**

**Background information of carer**

Age : ____________
Gender : ____________
Ethnicity : ____________
Marital statue : Single/ Married/ Divorced/ Separated
Highest edcucational level: Primary/Secondary/ Tertiary
Occupation : ______________________
Estimated family income : RM _________ /month

**Background information of child**

Age of child:___________(year)
Gender: _________________________
Who takes of your child most of the time (more than 12 hours a day) :
You/ your partner/ child's grandmother or grandfather/ helper/ baby sitter
Others: _________________________________________________
How long has your child had asthma: _______________ (month/year)
Family members with asthma: Self/partner/children/siblings
Others: __________
