# Supplementary material for: How young children learn independent asthma self-management: a qualitative study in Malaysia
Source: Arch Dis Child. 2020 Jul 3;105(9):819–24. doi: 10.1136/archdischild-2019-318127 (PMC7456543; doi:10.1136/archdischild-2019-318127)
Supplement: Supplementary data [file archdischild-2019-318127supp002.pdf]

Childhood Asthma
Topic Guide for interviews and focus groups (children)

Preamble:

- **Ice-breaking and explain that there is no right or wrong answer**
- **Explain need to get consent for the interview and audio-recording. Obtain consents.**
- **Explain that the participant does not have to answer if he or she does not wish to do so**

Question	Prompts
**Asthma experience**	
What do you know about asthma?	Experience? Reading? Told by someone?
Tell us about your asthma? How is it now?	What do you feel? Cough, nightime cough, difficulty breathing?
How do you know if it's good or bad asthma?	Able to play? Easy breathing? No cough?<br>**Where do you know about this?**
How does your asthma disturb/not disturb you?	Activities affected e.g school? swimming, PJK, sleeping in fan?air conditioned room? Early morning or night bath? Play in the rain?
How does having asthma makes you feel?	Friends isolation/bully- because of illness, using inhalers, Shy?
**Asthma self-management**	
What do you do when you have your asthma?	Inhaler? Tell parents? Go to hospital/clinic? Asthma action plan? **How do you know this?**
Who take care of your asthma at home? School?	You? Your parents? Maid? What do you/they do?
Do you take anything else other than these for your asthma? (showing inhalers and syrup)	Other medication from doctors?
**Asthma treatment**	
Do you take medicine for asthma?<br>Tell us about your asthma medicine.	Pills? MDI? Spacer? Aerochamber? Have you seen 'these' (peak flow meter) before? How do you use it?<br>When do you use your medication? **How often?**<br>Which medication is better? Reliever ? controller?<br>How would you know when do you need the pump?
How do you feel about taking asthma medicines?	Dislike? Troublesome? Ashamed? **Why?**
How often do you see the doctor for asthma?	Follow up? Emergency (hospital)? Stay in hospital ? When? Why?

When you go to clinic what was usually done?<br><br>What do you think about the clinic visits for asthma (clinic, doctor & medications)?<br><br>How do you feel when you come to clinic?	Explanation? Medication?<br><br>Difficult access? Language barrier? Waiting time? Unfriendly staff?<br><br>Unpleasant? Okay?
**Health belief about asthma**<br><br>Why do you think you have asthma?<br><br>**What triggers your child's asthma?**<br><br>What other things you do/eat for your asthma?<br><br>Do you have rules about asthma?	Inherited? Infection?<br><br>**Dust? Physical activity? Cold weather?**<br><br>What are they? Home nebulizer? How does it help? Homeopathy? Which method is better for you? Why? **Where did you know this from?**<br><br>**What do you usually eat?**<br><br>Food, drinks or activities? Any other rules?<br><br>Who makes the rules? (rules at school and home) **Experience? Or from somewhere? What will happen if you break this rule?**
**Source of information**<br><br>Where and from whom did you get information about asthma?<br><br>Anything else that you want to share about your asthma?	Clinic? Family? Friends? Internet? How good is the information?

Childhood Asthma
Topic Guide for interviews and focus groups (carers)

Preamble:
- **Ice-breaking and explain that there is no right or wrong answer**
- **Explain need to get consent for the interview and audio-recording. Obtain consents.**
- **Explain that the participant does not have to answer if he or she does not wish to do so**

Question	Prompts
**Asthma experience**<br><br>What do you know about asthma?<br><br>Tell us about your child's asthma? How is it now?<br><br>How does your child's asthma disturb/not disturb he/her? How? How does your child's asthma disturbs/not disturb you? How?<br><br>How do you know if your child's asthma is good or bad?<br><br>How does having asthma makes your child feel?<br><br>How does your child's asthma makes you feel?	Experience? Reading? Told by someone?<br><br>Cough, nightime cough, difficulty breathing?<br><br>Activities affected e.g school? swimming, PJK, sleeping in fan? air conditioned room? Early morning or night bath? Play in the rain?<br><br>Able to play? Easy breathing? No cough?<br><br>Friends isolation/bully- because of illness, using inhalers, shy?<br><br>Anxious? Overprotective?
**Asthma self-management**<br><br>What do you do when your child has asthma?<br><br>Who take care of your child's asthma at home? School?<br><br>Does your child take anything else other than these for his/her asthma? (showing inhalers and syrup)	Inhaler? Go to hospital/clinic? Asthma action plan?<br><br>You? Your child? Maid? What do you/they do?<br><br>Other medication from doctors?
**Asthma treatment**<br><br>Does your child take medicine for asthma?<br><br>Tell us about your child's medicine.	Pills? MDI? Spacer? Aerochamber? Have you seen 'these' (peak flow meter) before? How do you use it?<br><br>When do you use your medication?<br><br>Do you need to take your medicine everyday?<br><br>Which medication is better? Reliever ? controller?<br><br>How would you know when do your child

	needs the pump?
How do you feel about your child taking medicines?	Dislike? Troublesome? Ashamed?
How often do your child see the doctor for asthma?	Follow up? Emergency (hospital)? Stay in hospital ? When? Why?
When your child goes to clinic what was usually done?	Explanation? Medication?
What do you think about the clinic visits for asthma (clinic, doctor & medications)?	Difficult access? Language barrier? Waiting time? Unfriendly staff?
How do you feel when you accompany your child to clinic?	Unpleasant? Okay?
**Health belief about asthma**	
Why do you think your child has asthma?	Inherited? Infection? Environment?
What other things you do/eat for your child's asthma?	What are they? Home nebulizer? How does it help? Homeopathy? Which method is better for you? Why?
Do you have rules about asthma?	Food, drinks or activities? Any other rules?
	Who makes the rules? (rules at school and home)
**Source of information**	
Where and from whom did you get information about asthma?	Clinic? Family? Friends? Internet? How good is the information?
Anything else that you want to share about your child's asthma?	
